# Biochemical, histopathological, and immunohistochemical study on the ameliorative effect of crocin against lipopolysaccharide‑induced hippocampal toxicity in male albino rats

**DOI:** 10.1186/s40360-025-01021-y

**Published:** 2025-11-25

**Authors:** Eatemad A. Awadalla, Ola Mohamed, Ahmed Abdelsadik, Hoda S. Sherkawy, Abd El-Kader M. Abd El-Kader

**Affiliations:** 1 Department of Zoology, Faculty of Science, Aswan University, Aswan, 81511 Egypt; 2 Department of Medical Biochemistry, Faculty of Medicine, Aswan University, Aswan, 81511 Egypt

**Keywords:** Lipopolysaccharide, Crocin, Captopril, Hippocampal toxicity, Memory impairments

## Abstract

**Background:**

Lipopolysaccharide (LPS)-induced neuroinflammation is widely used as an animal model for studying the mechanisms of neuroinflammation. Crocin, an active component of saffron (*Crocus sativus L*), possesses several beneficial properties. The present study aimed to investigate the role of crocin in alleviating hippocampal toxicity induced by LPS in rats.

**Method:**

Forty male albino rats were randomly divided into five groups. Group I served as a control. Group II intraperitoneally (i.p.) injected with LPS (1 mg/kg/day) for a week. Groups III, IV, and V were treated by oral gavage with captopril (50 mg/kg/day), crocin (50 mg/kg/day), and a combination of both captopril (50 mg/kg/day) and crocin (50 mg/kg/day), respectively for 30 consecutive days, starting on the 8th day after LPS i.p. injection. During the therapy schedule, rats were tested for memory and learning abilities. Hippocampal samples were collected for biochemical, histological, immunohistochemical, and morphometric studies. Biochemical evaluation included nuclear factor kappa B, inflammatory cytokines (tumor necrosis factor-α and interleukin-1β), amyloid beta, angiotensin-converting enzyme, markers of the cholinergic system (acetylcholinesterase and choline acetyltransferase), antioxidant enzymes (catalase and superoxide dismutase) and an oxidative stress indicator (malondialdehyde). Histological examinations, as well as immunohistochemical and histomorphometric analysis, were also performed on hippocampal tissue.

**Results:**

The results revealed biochemical, histological, and immunohistochemical alterations in the hippocampus of the LPS group. Most of these alterations showed satisfactory improvements in hippocampal tissue when LPS-administered rats were treated with captopril and crocin, either separately or in combination.

**Conclusion:**

The present study suggests that crocin acts as a promising therapeutic agent for alleviating memory impairments and neuroinflammation induced by LPS.

## Background

Lipopolysaccharide (LPS), a key gram-negative bacteria cell wall component, is extensively utilized in experimental models of amyloidosis and neuroinflammation [1], affecting various neurological disorders including Alzheimer’s disease (AD) [2], Parkinson’s disease [3], amyotrophic lateral sclerosis [4], and multiple sclerosis [5]. The exact cause of AD remains unknown, despite various hypotheses including neuroinflammation, the amyloid beta (Aβ) deposition, and cholinergic hypothesis [6].

Nuclear factor kappa B (NF-κB), a key inflammation regulator, is extensively researched [7]. Bacterial toxins, like LPS, are believed to trigger NF-κB activation, implicating it in AD progression [8, 9].

LPS enters the brain and impacts the central nervous system through interactions with cluster of differentiation (CD14) on microglial membranes, forming the LPS-CD14 complex and binding to Toll-like receptor 4 (TLR4), activating microglia and initiating signal transduction cascades [10, 11]. NF-κB activation triggers gene transcription, releasing proinflammatory cytokines like tumor necrosis factor-α (TNF-α) and interleukin- 1β (IL-1β), which increase angiotensin-converting enzyme (ACE) levels [12] and promote AD pathogenesis by increasing amyloid precursor protein expression and Aβ formation [13].

LPS is also associated with the overproduction of reactive oxygen species (ROS), which overwhelms the brain’s antioxidant defenses. This imbalance leads to lipid peroxidation, causing oxidative stress and apoptotic neuronal death [14].

Captopril has been shown to reduce microglial activation and amyloid burden in AD models, indicating its role in mitigating neuroinflammation. In vivo studies demonstrated that intranasal administration of captopril significantly decreased markers of inflammation and amyloid plaques in transgenic mice [15]. Captopril treatment in aged Tg2576 mice resulted in normalized ACE activity and reduced reactive oxygen species (ROS), which are linked to neurodegeneration. Also, the drug’s ability to decrease amyloidogenic processing of amyloid precursor protein (APP) further supports its potential in slowing AD progression [16]. Studies have shown that captopril can enhance memory function and increase brain-derived neurotrophic factor (BDNF) expression in experimental models of AD. This suggests that captopril not only addresses neuroinflammation but also supports cognitive functions, which are often impaired in AD patients. While the evidence is promising, it is essential to recognize that the understanding of captopril’s full impact on AD is still developing, and further clinical studies are necessary to establish definitive therapeutic roles [17]. Captopril attenuates oxidative stress and neuroinflammation implicated in cisplatin-induced cognitive deficits in rats [18]. In parallel, increasing attention has been directed toward bioactive compounds from medicinal plants, which have shown therapeutic potential in various neurological and systemic disorders [19]. Crocin, one of the major carotenoid compounds derived from saffron [20, 21], has anti-inflammatory, antioxidant, and memory-enhancing properties, making it beneficial for treating nervous system disorders such as AD [22, 23]. Crocin (CR) reduced the levels of NF-κB and phosphorylated NF-κB in crocin-treated animals. This effect was attributed to the suppression of seizure-induced ROS generation and the associated NF-κB pathway-linked neuronal damage [24]. CR was shown to inhibit microglial activation and decrease the expression of proinflammatory mediators, such as IL-1β and TNF-α, induced by LPS in both in *vitro* and in *vivo* models [25]. CR administration to aged rats has been shown to decrease acetyle cholinesterase (AChE) activity and increased acetylcholine (ACh) level, resulting in memory function improvement [26]. Also, crocin’s protective effect against cognitive memory impairment is related to its ability to reduce Aβ formation [27].

Therefore, we decided to test the effects of captopril and crocin on lipopolysaccharide-induced learning and memory impairments, cholinergic system dysfunction, oxidative damage, and neuroinflammation in brain tissues of male albino rats.

## Materials and methods

### Materials

LPS, captopril (CAP), and CR were obtained from Sigma- Aldrish Co., USA, with CAS numbers 17,304, 441,600 and 2630, respectively. Commercial ELISA kits of TNF-α, IL-1β, NF-κB, Aβ, ACE, and choline acetyltransferase (ChAT) were purchased from Bioassay Technology Laboratory, Egypt, with catalog numbers E0082Hu, E0143Hu, E0690Hu, E1264Hu, E0927Hu, and E5039Hu, respectively. Catalase (CAT), superoxide dismutase (SOD), and lipid peroxide (MDA) were measured using commercial colorimetric kits purchased from Bio Diagnostics, Egypt, with catalog numbers CA 25 17, SD 25 21, and MD 25 29, respectively. Acetylthiocholine iodide and dithiobisnitrobenzoate (DTNB) were purchased from Alfa Aesar Co., China. All other chemicals were of the highest quality available.

### Ethical statement

The Animal Ethical Committee of Aswan University in Aswan, Egypt, approved all experimental methods, which were conducted in compliance with regional institutional policies. The ethical approval code is ASWU/05/SC/ZO/24 − 01/0.

### Animals and experimental design

Forty male albino rats weighing 140 ± 20 g were obtained from the Animal House, Faculty of Science, South Valley University. Animals were kept in a well-ventilated and clean cages and maintained under a 12 h:12 h schedule of light: dark cycle at 25 ± 2 °C with a relative humidity of 15 ± 5% at the Animal House, Faculty of Science, Aswan University. Rats were housed for approximately two weeks before the study began for acclimatization.

The rats were randomly split into five equal groups of eight rats. The groups were assigned according to the treatment as follows:

Control group: The rats were not treated with any material.

LPS group: The rats were injected intraperitoneally (i.p.) with LPS (1 mg/kg b.w.) for a week [28].

LPS + CAP group: Following a week of LPS injection, this group was treated with CAP (50 mg/kg b.w.) by oral gavage for 30 days [28].

LPS + CR group: Following a week of LPS injection, this group was treated with CR (50 mg/kg b.w.) by oral gavage for 30 days [29].

LPS + CAP + CR group: Following a week of LPS injection, this group was treated with a combination of CAP and CR by oral gavage for 30 days.

####  Note 

LPS was freshly dissolved in normal saline, and both CR and CAP were freshly dissolved in distilled water.

### Experimental procedures

24 h after the last dose, rats of the different groups were anesthetized using isoflurane inhalation and then animals were sacrificed by decapitation. Brains were immediately dissected on an ice-cold plate, and the hippocampus was isolated and divided into two parts, one part was stored at -80 °C for biochemical assays, and the other part was used for histological investigation. For biochemical assays we used Teflon-glass manual homogenizer. For sample preparation, a tissue sample of 0.1 g was placed in a pre-chilled tube and homogenized with 1 mL of cold phosphate-buffered saline (PBS, pH 7.4). Homogenization was performed manually on ice using approximately 15 complete strokes for 1–2 min to ensure proper breakdown of the tissue while minimizing protein degradation. Then, the homogenate was centrifuged at 4,000 g for 10 min at 4 °C, and the supernatant was collected for subsequent biochemical analysis.

## Behavioral tests

### Novel object recognition test

The current study used open-field test box (45 × 45 × 50 cm) to perform a novel object recognition test [30]. All animals were habituated to the test box for 10 min without the presence of objects. Following the habituation period, each animal was placed in the test box with two identical objects and allowed to explore for 5 min. The time spent by each rat exploring the objects was recorded. After a 24-hour delay, the animals were reintroduced to the test arena for 5 min, which now contained one of the original objects from the training session and one novel object. The discrimination ratio (DR) was calculated as the difference in exploration time between the novel (N) and familiar (F) objects, divided by the total exploration time (ET): DR = (N − F) / (N + F). The recognition index (RI) was calculated as the exploration time of the novel object divided by the total exploration time: RI = N / (N + F).

### T-maze

The T-maze test was conducted using a discrete trial procedure, as described by Grzeda et al. [31]. Spontaneous alternation relies on the rat’s natural tendency to explore new environments by alternating between goal arms of the maze. The apparatus consisted of a wooden T-maze with a start arm (15 × 50 cm) and two perpendicular goal arms (10 × 50 cm). At the beginning of each trial, the rat was placed at the start arm facing away from the goal arms. A choice was recorded once all four paws entered a goal arm. The selected arm was then blocked, and the rat remained there for 30 s before being removed and returned to the start position for the next trial. An alternation was considered correct when the rat entered the arm opposite to the one previously visited. The percentage of alternation was calculated as the ratio of correct alternations to the total number of trials.

### Enzyme-linked immunosorbent assay (ELISA)

Determination of Biomarkers (NF-κB, TNF-α, IL-1β, Aβ, ChAT, and ACh).

The levels of nuclear factor kappa B (NF-κB), tumor necrosis factor-α (TNF-α), interleukin-1β (IL-1β), amyloid-β (Aβ), choline acetyltransferase (ChAT), and acetylcholine (ACh) in hippocampal homogenates were determined using ELISA kits (Bioassay Technology Laboratory, Egypt) following the manufacturer’s protocols. Briefly, standards or samples were added to the wells, incubated with the corresponding specific antibody and streptavidin-HRP, and then treated with substrate solutions. The reaction was stopped, and absorbance was measured at 450 nm using a microplate reader.

### Calculation of results

Concentrations of each biomarker were determined from standard curves generated by plotting optical density (OD) values against known concentrations, with regression analysis used to calculate sample values.

### Estimation of acetylcholinesterase (AChE) levels

AChE activity in hippocampal homogenates was measured using Ellman’s method [32]. The assay is based on hydrolysis of acetylthiocholine to thiocholine, which reacts with DTNB to form a yellow product measured at 412 nm.

### Procedure

Fifty µL of sample was mixed with phosphate buffer (pH 8), acetylthiocholine iodide, and DTNB. Absorbance was recorded at 412 nm for 2 min, and activity was expressed as nmol/min/mg tissue.

## Spectrophotometry

### Catalase (CAT) activity

Measured according to the method of Aebi [33]. Tissue homogenates were prepared in phosphate buffer (50 mM, pH 7.0). The assay mixture contained H₂O₂ (30 mM) in phosphate buffer, and the decomposition rate of H₂O₂ was monitored spectrophotometrically at 240 nm for 1 min. One unit of CAT activity was defined as the amount of enzyme decomposing 1 µmol of H₂O₂ per minute, expressed as U/mg protein using the molar extinction coefficient of H₂O₂ (43.6 M⁻¹cm⁻¹).

### Superoxide dismutase (SOD) activity

Assayed following the method of Beauchamp and Fridovich [34]. The assay system consisted of 50 mM phosphate buffer (pH 7.8), 0.1 mM EDTA, 13 mM methionine, 75 µM nitroblue tetrazolium (NBT), and 2 µM riboflavin. Samples were illuminated under fluorescent light for 10 min, and absorbance was read at 560 nm. One unit of SOD activity was defined as the amount of enzyme required to inhibit NBT reduction by 50%, and the results were expressed as U/mg protein.

### Malondialdehyde (MDA) levels

Estimated by the thiobarbituric acid reactive substances (TBARS) assay according to Ohkawa et al. [35]. Tissue homogenates were mixed with thiobarbituric acid (0.67%) and trichloroacetic acid (10%) and heated at 95 °C for 30 min. After cooling and centrifugation, absorbance was measured at 532 nm. MDA concentration was calculated using the extinction coefficient (1.56 × 10⁵ M⁻¹cm⁻¹) and expressed as nmol MDA/mg protein.

#### Histological examinations

The hippocampal samples were fixed in 10% neutral buffered formalin, dehydrated through a graded series of alcohols, cleared in methyl benzoate, and embedded in paraffin wax at 58–62 °C. Sections of 5 μm thickness were cut and stained with Harris’ hematoxylin and eosin stain [36]. Microscopic examination was performed to describe the general histological architecture of the hippocampus. The observations focused on morphological alterations such as nuclear pyknosis, neuronal shrinkage, vacuolation, cellular atrophy and proliferation of glial cell. The findings were documented qualitatively and illustrated with representative photomicrographs.

### Immunohistochemical examinations

The expression of caspase-3 and interleukin − 6 (IL-6). According to ABclonal kit method (ABclonal Technology Company, USA), formalin-fixed, paraffin-embedded hippocampal tissue slices were sectioned at a thickness of 4–5 μm. The sections were deparaffinized and rehydrated. After applying the antigen retrieval solution, endogenous peroxidase activity was inactivated for 15 min using 3% hydrogen peroxide, and then the sections were blocked for an hour. The rabbit polyclonal primary antibody (2.5% normal goat serum diluted in PBS/TBS supplemented with 0.3% Triton™ X-100, pH 7.2) for caspase-3 (ab4051) and for IL-6 (Cell Signaling, 12153 S) was applied to each section and incubated overnight in a humidified chamber at 4 °C. Then, after applying the peroxidase-labeled secondary antibody [HRP goat anti-rabbit IgG (H + L)] (Dako, EnVision FLEX, High 800021 at a 1:200 dilution), the samples were incubated for 30 min. Freshly prepared DAB substrate and chromogen (Dako, EnVision FLEX, High pH (Link) kit # K800021) were used at room temperature for 2–5 min. Hematoxylin was used as a counterstain; sections were dried and then mounted.

### Histomorphometric study and image analysis

Histomorphometric analysis was used to quantify structural changes observed in the histological analysis of hippocampal tissue. After the hippocampus was histologically processed, digital images were taken at 40× magnification using a digital camera connected to a light microscope (Olympus BX43F, Olympus Corporation, Shinjuku-ku, Tokyo, Japan). Histomorphometric analysis was performed using the ImageJ software system, version 6. Spatial calibration with an object micrometer was conducted before each analysis. Five images were selected from each animal in each group. The following morphometric parameters were measured: thickness of the pyramidale layer, % caspase-3 intensity/surface area, and % interleukin-6 intensity/surface area.

### Statistical analysis

All quantitative data were expressed as mean ± deviation (SD). For histological, behavioral, and biochemical analyses (except AChE), the data were normally distributed but showed unequal variances; therefore, statistical comparisons were performed using Welch’s ANOVA and Brown–Forsythe ANOVA. For acetylcholinesterase (AChE) activity, which did not follow a normal distribution, the Kruskal–Wallis test was applied as a non-parametric alternative to one-way ANOVA. A p-value < 0.05 was considered statistically significant.

## Results

### Behavioral tests

The values of the discrimination ratio (DR) and recognition index (RI) in the novel object recognition test, as well as the % alternation in the T-maze, showed a highly significant decrease in the LPS group (0.207 ± 0.013, 0.46 ± 0.015, and 38.47 ± 0.016, respectively; *P* < 0.01 for all) compared with the control group (0.743 ± 0.022, 0.852 ± 0.020, and 83.71 ± 0.025, respectively). However, the values in the LPS + CAP group exhibited a significant increase (0.438 ± 0.024, 0.617 ± 0.022, and 53.56 ± 0.012, respectively; *P* < 0.05 for all) compared with the LPS group. Additionally, the values in the LPS + CR group (0.571 ± 0.011, 0.763 ± 0.012, and 73.12 ± 0.017, respectively; *P* < 0.001 for all) and in the LPS + CAP + CR group (0.644 ± 0.017, 0.812 ± 0.016, and 81.42 ± 0.015, respectively; *P* < 0.001 for all) showed a highly significant increase compared to those in the LPS group (Table 1).

**Table 1:** Behavioral tests results, including discrimination ratio (DR), recognition index (RI), and % alternation in various study groups

Groups parameters	control	LPS	LPS + CAP	LPS + CR	LPS + CAP + CR
DR	0.734 ± 0.134	0.207 ± 0.041^*^	0.438 ± 0.052^#^	0.571 ± 0.094^##^	0.644 ± 0.041^##^
RI	0.852 ± 0.068	0.46 ± 0.073^*^	0.617 ± 0.055^#^	0.763 ± 0.074^##^	0.812 ± 0.068^##^
% alternation	83.71 ± 4.786	38.47 ± 6.821^*^	53.56 ± 4.948^#^	73.12 ± 9.399^##^	79.09 ± 7.608^##^

Values are expressed as mean ± SD for animals in each group

* Highly significant compared with the control group (*P* < 0.01)

# Significantly different compared with the LPS group (*P* < 0.05)

## Highly significant compared with the LPS group (*P* < 0.001)

### Enzyme-linked immunosorbent assay (ELISA)

### The nuclear factor kappa B (NF-κB), tumor necrosis factor-α (TNF-α), Interleukin-1β (IL-1β), amyloid beta (Aβ) in various study groups

Values of NF-κB, TNF-α, IL-1β, and Aβ concentrations in the LPS group showed a highly significant increase (2.612 ± 0.171 ng/ml, 3.143 ± 0.026 ng/l, 6.626 ± 0.128 pg/ml, and 3.79 ± 0.014 ng/l, respectively; *P* < 0.001 for all) compared with the control group (0.762 ± 0.06 ng/ml, 1.093 ± 0.014 ng/l, 2.89 ± 0.018 pg/ml, and 1.006 ± 0.032 ng/l, respectively). Additionally, the values in the LPS + CAP-treated animals were found to be significantly decreased (1.847 ± 0.012 ng/ml, 2.13 ± 0.021 ng/l, 5.054 ± 0.107 pg/ml, and 2.087 ± 0.025 ng/l, respectively; *P* < 0.01 for all) compared with the LPS group. Meanwhile, there was a highly significant reduction in the LPS + CR group (1.229 ± 0.013 ng/ml, 1.684 ± 0.031 ng/l, 4.07 ± 0.015 pg/ml, and 1.564 ± 0.018 ng/l, respectively; *P* < 0.001 for all) and in the LPS + CAP + CR group (0.897 ± 0.021 ng/ml, 1.261 ± 0.011 ng/l, 3.057 ± 0.014 pg/ml, and 1.272 ± 0.027 ng/l, respectively; *P* < 0.001 for all) compared with the LPS group (Table 2).

**Table 2:** NF-κB, TNF-α, IL-1β, and Aβ in various study groups

Groups parameters	Control	LPS	LPS + CAP	LPS + CR	LPS + CAP + CR
NF-κB (ng/ml)	0.762 ± 0.019	2.567 ± 0.459^*^	1.988 ± 0.007^#^	1.134 ± 0.036^##^	0.973 ± 0.032^##^
TNF-α (ng/l)	1.093 ± 0.201	3.143 ± 0.539^*^	2.13 ± 0.585^#^	1.684 ± 0.536^##^	1.261 ± 0.421^##^
IL-1β (pg/ml)	2.89 ± 0.737	6.626 ± 1.114^*^	5.054 ± 0.783^#^	4.07 ± 0.604^##^	3.057 ± 0.561^##^
Aβ (ng / l)	1.006 ± 0.129	3.79 ± 0.401^*^	2.087 ± 0.636^#^	1.514 ± 0.456^##^	1.272 ± 0.355^##^

Values are expressed as mean ± SD for animals in each group

* Highly significant compared with the control group (*P* < 0.001)

# Significantly different compared with the LPS group (*P* < 0.01)

## Highly significant compared with the LPS group (*P* < 0.001)

### Angiotensin- converting enzyme (ACE) and choline acetyltransferase (ChAT) levels in various study groups

ACE and ChAT levels. A highly significant increase in ACE activity (1.248 ± 0.118 U/L tissue; *P* < 0.001) and a highly significant decrease in ChAT activity (1.13 ± 0.019 ng/L; *P* < 0.001) were observed in the LPS group compared to the control group (0.376 ± 0.02 U/L tissue for ACE and 4.39 ± 0.024 ng/L for ChAT). However, the activity of ACE in the LPS + CAP group was significantly decreased (0.871 ± 0.032 U/L tissue; *P* < 0.05), while the activity of ChAT was significantly increased (2.34 ± 0.128 ng/L; *P* < 0.05) compared with the LPS group. Meanwhile, the ACE activity was found to be highly significant reduction in the LPS + CR group (0.653 ± 0.014 U/L tissue; *P* < 0.001) and in the LPS + CAP + CR group (0.407 ± 0.012 U/L tissue; *P* < 0.001) compared with the LPS group. Additionally, the values of ChAT levels in the LPS + CR group and the LPS + CAP + CR group showed a highly significant increase (3.05 ± 0.011 ng/l and 4.09 ± 0.002 ng/l, respectively; *P* < 0.001 for both) compared with LPS- administered animals (Table 3).

**Table 3:** ACE, AChE, and chat levels in various study groups

Groups parameters	Control	LPS	LPS + CAP	LPS + CR	LPS + CAP + CR
ACE	0.376 ± 0.064	1.248 ± 0.486^*^	0.871 ± 0.113 ^#^	0.654 ± 0.078^##^	0.407 ± 0.084^##^
AChE	0.749 ± 0.069	2.193 ± 0.47^*^	1.429 ± 0.562^#^	1.084 ± 0.383^##^	0.801 ± 0.101^##^
ChAT	4.39 ± 0.067	1.13 ± 0.026^*^	2.34 ± 0.643^#^	3.05 ± 0.025^##^	4.09 ± 0.245^##^

Values are expressed as mean ± SD for animals in each group

* Highly significant compared with the control group (*P* < 0.001)

#Significant compared with the LPS group (*P* < 0.05)

## Highly significant compared with the LPS group (*P* < 0.001)

### Acetyle cholinesterase (AChE) level

As shown in Table 3, the results indicate that AChE activity in the LPS group was significantly higher (2.193 ± 0.117 nmol/mg tissue, *P* < 0.001) than in the control group (0.749 ± 0.0232 nmol/mg tissue). Furthermore, AChE activity in the LPS + CAP group was significantly lower (1.429 ± 0.021 nmol/mg tissue, *P* < 0.05) than in the LPS group. However, AChE activity in the LPS + CR group and the LPS + CAP + CR group showed a highly significant increase (1.084 ± 0.012 nmol/mg tissue and 0.801 ± 0.012 nmol/mg tissue, respectively; *P* < 0.001 for both) compared to that in the LPS group.

### Spectrophotometry. The levels of catalase (CAT), superoxide dismutase (SOD), and malondialdehyde (MDA) in various study groups

In LPS-administered animals, the CAT and SOD activities showed a highly significant decrease, while MDA levels showed a highly significant increase (3.05 ± 0.005 U/g and 164.01 ± 2.483 U/g for CAT and SOD, respectively, and 247.185 ± 0.881 nmol/g tissue for MDA; *P* < 0.001 for all) compared with control animals (9.841 ± 0.02 U/g, 363.47 ± 0.651 U/g, and 86.447 ± 0.11 nmol/g tissue, respectively). In the LPS + CAP group, CAT and SOD activity showed a significant increase, while MDA levels significantly decreased (5.196 ± 0.026 U/g and 245.63 ± 0.711 U/g for CAT and SOD, respectively, and 178.436 ± 0.166 nmol/g tissue for MDA; *P* < 0.01 for all) compared with the LPS group. However, CAT and SOD activities showed a highly significant increase, while MDA levels exhibited a highly significant decrease in both the LPS + CR group (6.94 ± 0.083 U/g and 320.05 ± 0.318 U/g for CAT and SOD, respectively, and 119.57 ± 1.116 nmol/g tissue for MDA; *P* < 0.001 for all) and the LPS + CAP + CR group (8.773 ± 0.007 U/g, 340.165 ± 0.109 U/g for CAT and SOD, respectively, and 91.248 ± 0.138 nmol/g tissue for MDA; *P* < 0.001 for all) compared with the LPS- administered animals (Table 4).

**Table 4:** CAT, SOD, and MDA levels in various study groups

Groups parameters	Control	LPS	LPS + CAP	LPS + CR	LPS + CAP + CR
CAT	9.841 ± 0.428	3.06 ± 0.895^*^	5.196 ± 0.741^#^	6.94 ± 0.912^##^	8.773 ± 0.738^##^
SOD	363.47 ± 51.28	164.01 ± 6.663^*^	245.63 ± 49.02^#^	320.05 ± 0.86^##^	340.165 ± 24.781^##^
MDA	86.447 ± 6.153	247.185 ± 43.225^*^	178.436 ± 14.043^#^	119.57 ± 2.99^##^	91.248 ± 6.411^##^

Values are expressed as mean ± SD for animals in each group

* Highly significant compared with the control group (*P* < 0.001)

#Significant compared with the LPS group (*P* < 0.01)

## Highly significant compared with the LPS group (*P* < 0.001)

### Histological findings of the hippocampus in various study groups

**Fig. 1:**
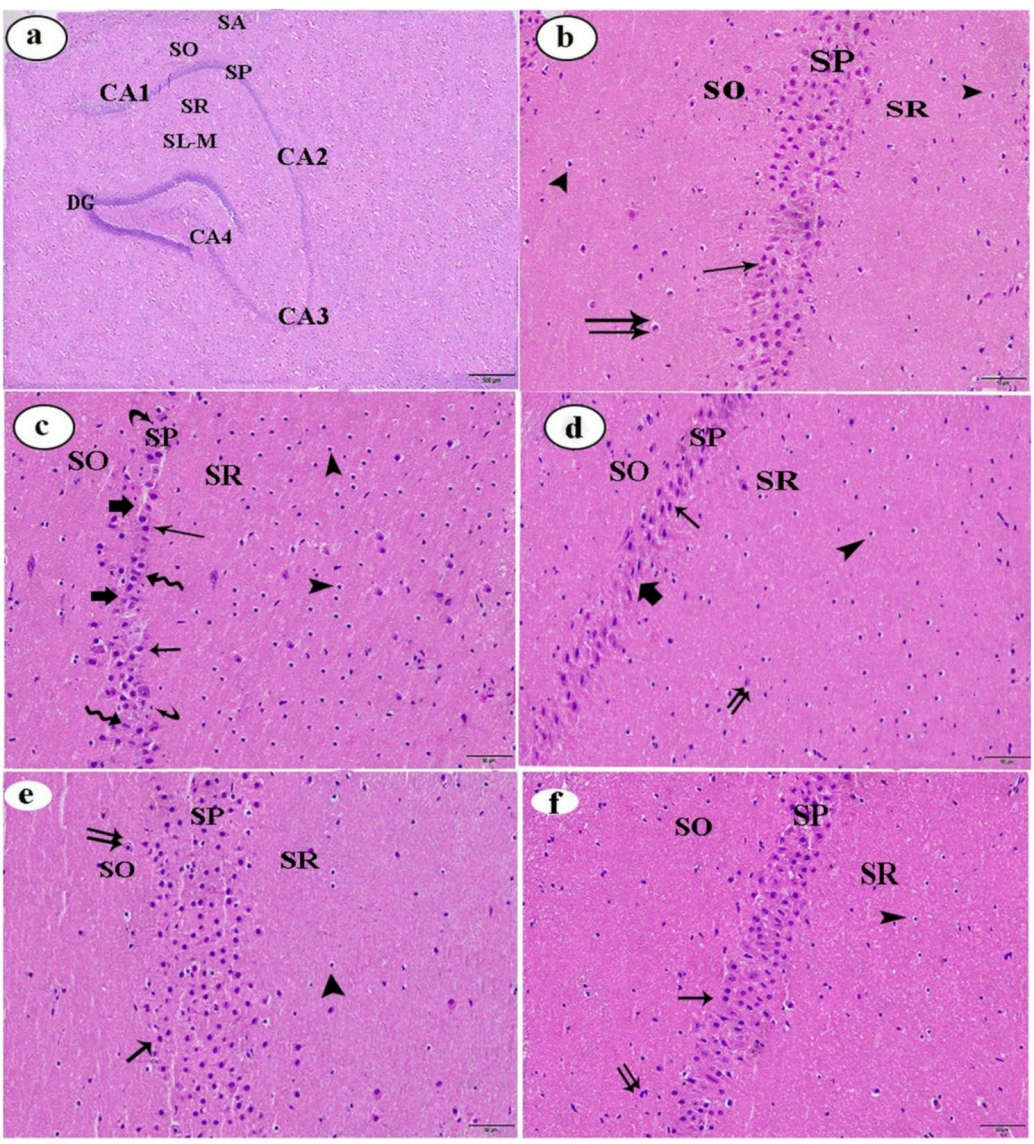
(**a**-**f**): Photomicrographs of hippocampal sections from male albino rats stained with H&E (original magnification: a, X200; b-f, X400). (**a** & **b**) Low and high magnifications of hippocampal sections from the control group. (**c**) Section from the LPS group. (**d**) Section from the LPS + CAP group. (**e**) Section from the LPS + CR group. (**f**) Section from the LPS + CAP + CR group. Stratum pyramidale (SP), stratum oriens (SO), stratum radiatum (SR). Pyramidal cells (thin arrow), neuroglia (arrowhead), blood capillaries (double arrow), dispersed vacuolization (thick arrow), shrunken neurons (curved arrow), atrophied and elongated cells (zigzag arrow). H&E: haematoxylin and eosin; LPS: lipopolysaccharide; CAP: captopril; CR: crocin

In the control group, histological analysis of the hippocampus revealed its characteristic bilateral, incurved, and seahorse-shaped structure, encompassing the cornu ammonis regions (CA1 and CA2, composed of small pyramidal cells) and (CA3 and CA4, composed of large pyramidal cells), as well as the dentate gyrus (DG). Each region of Cornu Ammonis consisted of five areas: stratum alveolus (SA), stratum oriens (SO), stratum pyramidale (SP), stratum radiatum (SR), and stratum lacunosum-moleculare (SL-M) (Fig. 1a). The SP of CA1 in the control group consisted of 4–5 layers of small pyramidal neurons. Neuroglial cells and blood capillaries with normal morphology were observed in the SO and SR (Fig. 1b). In contrast to the control group, hippocampal tissue from the LPS group exhibited severe histopathological alterations in the CA1 region. The SP showed significant vacuolization, reduced neuronal layers (2–3), pyknotic nuclei, neuronal shrinkage, cellular atrophy, and irregularly shaped cells. Gliosis was prominent in the SO and SR, indicating glial cell proliferation (Fig. 1c).

Compared with the LPS group, hippocampal tissue from the LPS + CAP group retained its normal appearance to some extent. In the CA1 region, the SP retained 2–3 discrete neuronal layers, with reduced pyknotic nuclei and decreased vacuolization. Moreover, moderate gliosis, indicated by diminished glial cell proliferation, was observed in the SO and SR (Fig. 1d). Histological investigation of the hippocampal sections from the LPS + CR and LPS + CAP + CR groups revealed substantial preservation of hippocampal architecture compared with the LPS group. The SP exhibited marked improvement, with neurons displaying normal morphology. Restoration of glial cell distribution and blood capillaries integrity was observed in the SO and SR (Fig. 1e and f, respectively).

### Immunohistochemical findings of the hippocampus in various study groups

### The expression of caspase-3 and interleukin-6 (IL-6)

**Fig. 2:**
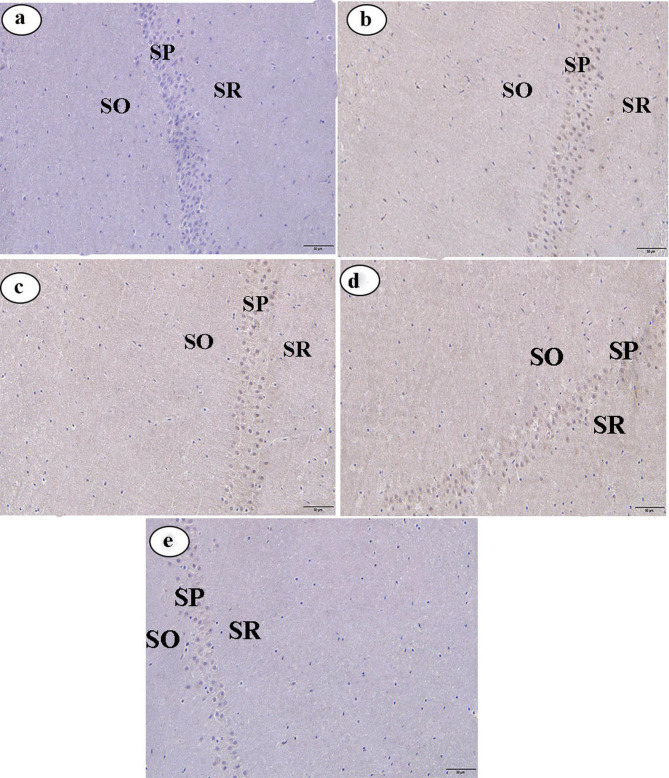
(**a**-**e**) Photomicrographs of hippocampal sections from male albino rats immunostained for caspase-3 (original magnification: X400). (**a**) Hippocampal section from the control group. (**b**) Section from the LPS group. (**c**) Section from the LPS + CAP group. (**d**) Section from the LPS + CR group. (**e**) Section from the LPS + CAP + CR group. Stratum pyramidale (SP), stratum oriens (SO), and stratum radiatum (SR)

**Fig. 3:**
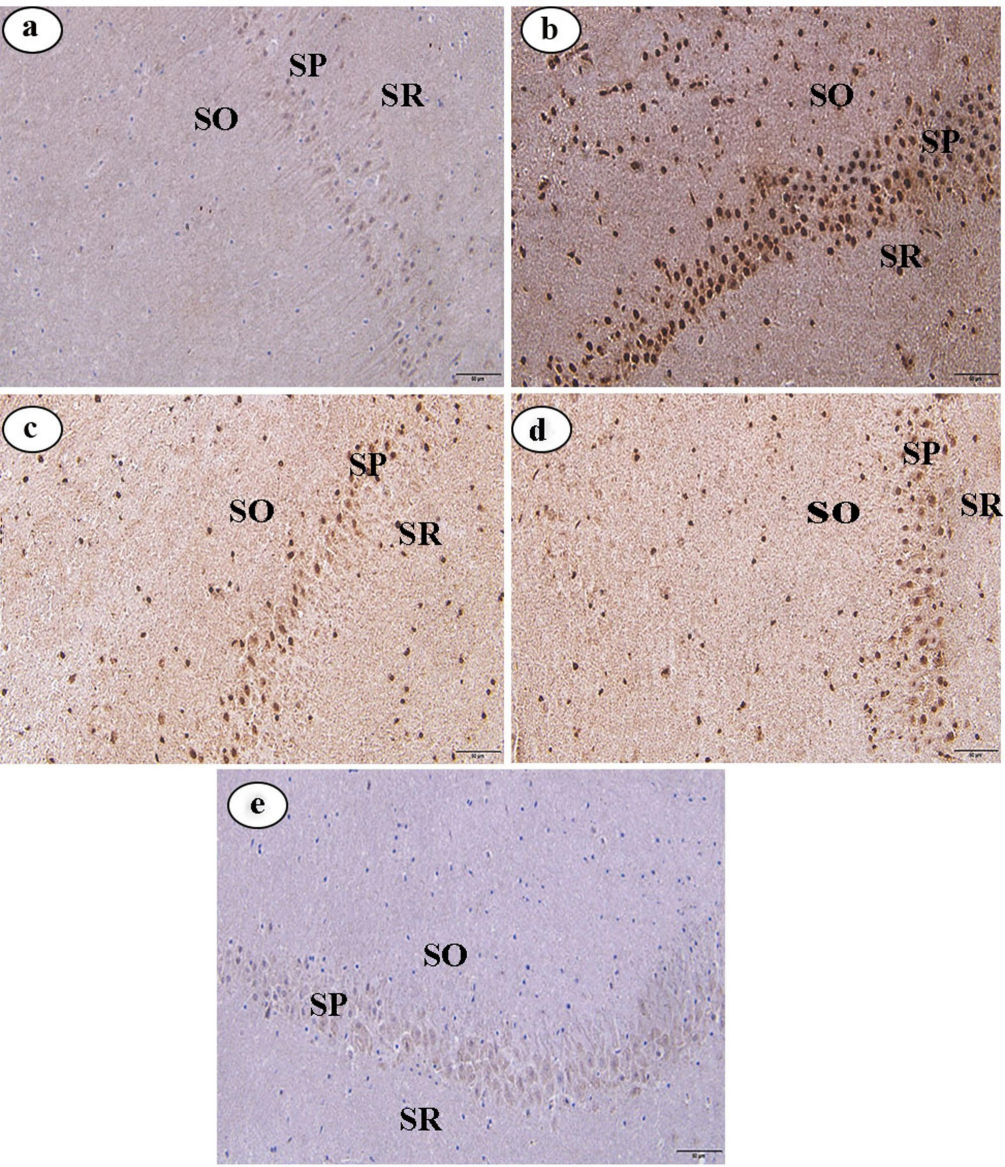
(**a**-**e**) Photomicrographs of hippocampal sections from male albino rats immunostained for IL-6 (original magnification: X400). (**a**) Hippocampal section from the control group. (**b**) Section from the LPS group. (**c**) Section from the LPS + CAP group. (**d**) Hippocampal section from the LPS + CR group. (**e**) Section from the LPS + CAP + CR group. Stratum pyramidale (SP), stratum oriens (SO), and stratum radiatum (SR)

In the control group, immunohistochemical analysis of caspase-3 and IL-6 expression in hippocampal tissues revealed minimal immunoreactivity, indicated by very weak brown staining in the SP, SO, and SR (Figs. 2a and 3a, respectively). Conversely, the LPS-administered group exhibited pronounced immunoreactivity for caspase-3 and IL-6, with intense brown staining in the same layers (Figs. 2b and 3b, respectively). Compared to the LPS-administered group, the LPS + CAP, LPS + CR, and LPS + CAP + CR groups exhibited weak caspase-3 and IL-6 immunoreactivity, as indicated by faint brown staining in the SP, SO, and SR of the CA1 region (Figs. 2c, d, and e, respectively, for caspase-3 expression, and Figs. 3c, d, and e, respectively, for IL-6).

### Histomorphometric findings of the hippocampus in various study groups

### Thickness of the SP of the CA1

**Fig. 4:**
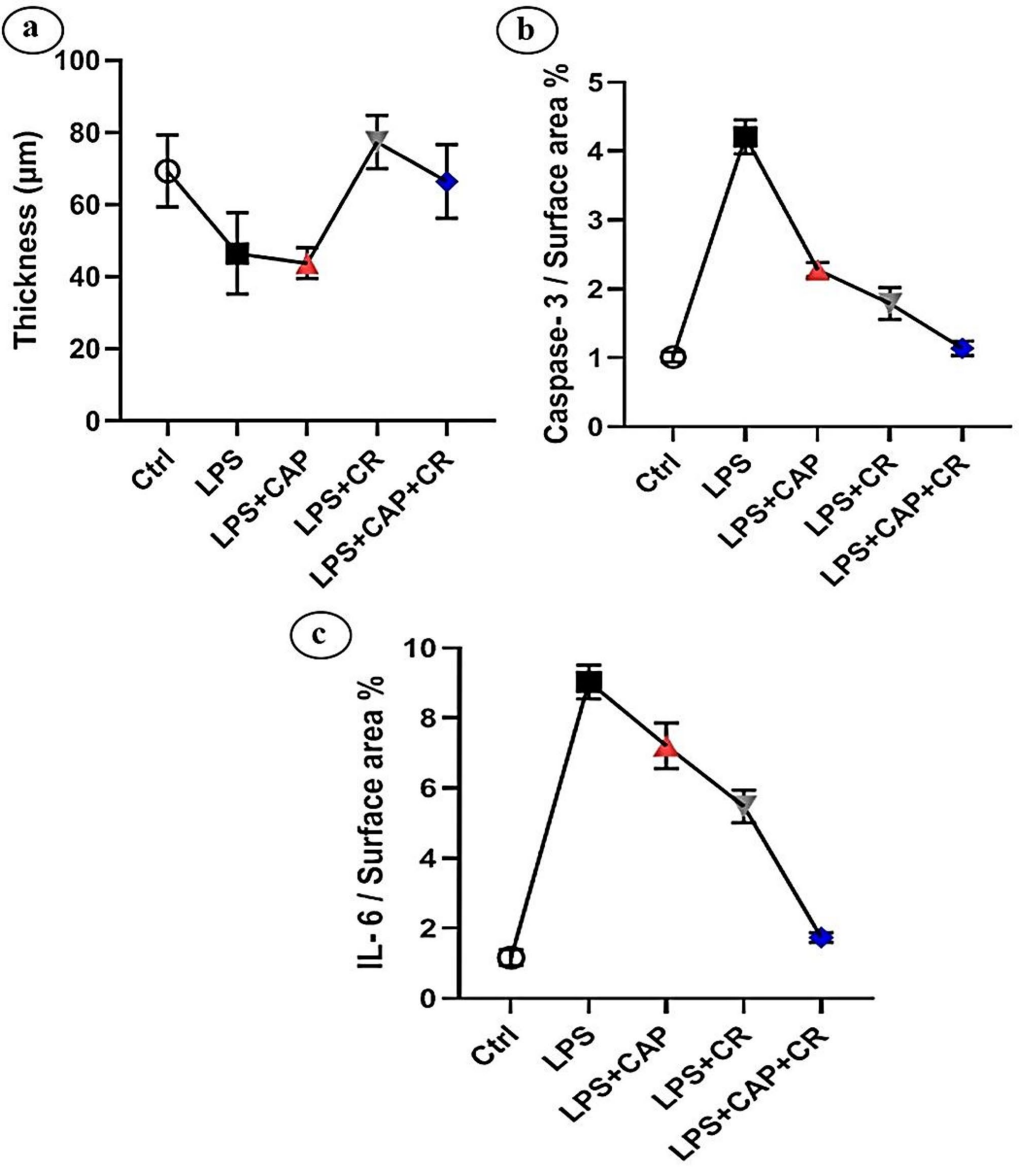
(**a**-**c**) Photomicrographs of morphometric results of the hippocampal sections of the rats in various study groups. (**a**) Histogram of the thickness of the SP of the CA1 region of all groups. (**b**) Histogram of the intensity color of immunohistochemical staining for caspase-3 in CA1 region of all groups. (**c**) Histogram of the intensity of immunohistochemical staining for IL-6 in CA1 region of all groups

There was a statistically significant decrease in the thickness of the SP of the CA1 in the LPS-administered group (46.50 ± 0.155 μm; *P* < 0.05) relative to the control group (69.38 ± 0.045 μm). Meanwhile, there was a non-significant change in the thickness of the SP of the CA1 (43.79 ± 0.291 μm; *P* > 0.05) in the LPS + CAP group relative to the LPS-administered group. Compared to the LPS group, the LPS + CR group and LPS + CAP + CR group showed a statistically highly significant increase in the thickness of the SP of the CA1 (77.42 ± 0.494 μm and 66.48 ± 0.199 μm, respectively; *P* < 0.001 for both) (Fig. 4a; Table 5).

**Table 5:** Morphometric results of the thickness of the SP area in the CA1 region and the intensity of immunohistochemical staining for caspase-3 and IL-6 in the CA1 region

Groups parameters	Control	LPS	LPS + CAP	LPS + CR	LPS + CAP + CR
Thickness	69.38 ± 10.03	46.49 ± 11.256^**^	43.79 ± 4.279^*^	77.41 ± 7.378^###^	66.48 ± 10.192 ^###^
Intensity of caspase-3	1.015 ± 0.076	4.21 ± 0.245^##^	2.283 ± 0.104^#^	1.789 ± 0.231^###^	1.14 ± 0.104^###^
Intensity of IL-6	1.17 ± 0.228	9.03 ± 0.479^##^	7.21 ± 0.649^#^	5.48 ± 0.463^#^	1.742 ± 0.138^###^

Values are expressed as mean ± SD for animals in each group

* Non-significant compared with the LPS group (*P* > 0.05)

** Significant compared with the control group (*P* < 0.01)

# Significant compared with the LPS group (*P* < 0.01)

## Highly significant compared with the control-group (*P* < 0.001)

### Highly significant compared with the LPS group (*P* < 0.001)

### Intensity of caspase-3 and IL-6 in the CA1 region of hippocampal sections

There was a highly significant increase in the intensity of caspase-3 and IL-6 in the CA1 region of hippocampal sections in the LPS group (4.210 ± 0.132 and 9.03 ± 0.154, respectively; *P* < 0.001 for both) compared to the control group (1.051 ± 0.341 and 1.17 ± 0.087, respectively). Conversely, the LPS + CAP group exhibited a significantly reduced staining intensity of caspase-3 and IL-6 in the CA1 region (2.283 ± 0.098 and 7.21 ± 0.201; *P* < 0.05 for both) compared to the LPS-induced group. Furthermore, the LPS + CR group showed a highly significant reduction in caspase-3 staining intensity (1.789 ± 0.214; *P* < 0.001) and a significant reduction in IL-6 staining intensity (5.48 ± 0.132; *P* < 0.05) relative to the LPS-treated group. However, the LPS + CAP + CR group exhibited a highly significant decrease in caspase-3 and IL-6 intensity in the CA1 region (1.14 ± 0.211 and 1.742 ± 0.102, respectively; *P* < 0.001 for both) (Fig. 4b and c, respectively; Table 5).

## Discussion

Behavioral tests are employed to evaluate their impact on cognitive functions, memory, and overall behavior. These tests help link the LPS-induced inflammation to behavioral changes, mainly in models of neurodegenerative diseases such as AD [37]. The present study demonstrated that the LPS group experienced a highly significant decrease in DR and RI in the novel object recognition task, as well as in alternation (%) in the T-maze test. The current results are reinforced by Frühauf et al. [38], Khan et al. [39], and Alshehri et al. [40], who explained that behavioral test performance was found to be reduced as a result of increased cytokine levels. In parallel with these results, reports from Bassi et al. [41], Okuyama et al. [42], and Lee et al. [43], who observed that LPS-induced changes in behavioral tests such as the novel object recognition task and T-maze. A possible explanation for these findings is the increase in LPS-induced neuronal cell death and the gradual neuronal loss, which lead to learning and memory dysfunction, as documented by Smith et al. [44] and Kranjac et al. [45].

The present findings in the LPS group revealed a highly significant elevation in the concentrations of NF-κB, TNF-α, IL-1β, Aβ, ACE and AChE activity, along with a highly significant reduction in ChAT levels in the homogenate of the brain hippocampus. Similarly, LPS activates NF-κB transcription factors, which play a critical role in various physiological processes, including synaptic plasticity related to learning and memory [43, 46, 47]. Similarly, when LPS binds to TLR4, triggering MyD88-dependent signaling, it activates the NF-κB pathway. NF-κB then translocates to the nucleus, inducing the transcription of proinflammatory cytokines such as IL-1β, IL-6, and TNF-α, thereby driving inflammation [48–50].

Evidence linking acute systemic inflammation, secondary neuroinflammation, and the accumulation of Aβ plaques in the brain is strengthened by the current study. The current findings are similar to those studies hypothesized that LPS-induced neuroinflammation impairs memory performance through elevated Aβ [51, 52].

ACE cooperates with LPS in the playing roles in the inflammatory pathways and contributes to neurodegenerative illnesses like AD [53]. Our observation is in agreement with the previous studies reported that LPS exposure triggers inflammatory pathways, including NF-κB signaling, and this response is linked to enhanced immune activity, contributing to elevated ACE levels [54, 55]. Additionally, our findings align with those of other researchers who have demonstrated that LPS administration activates localized renin angiotensin system (RAS) in the brain by increasing ACE levels [28, 56]. ACh is an excitatory neurotransmitter that plays an important role in memory, learning, and higher cognitive functions in the central cholinergic nervous system. ChAT regulates its synthesis and release, whereas AChE catalyzes its breakdown [57]. Additionally, in accordance with the present results, several supporting evidences have revealed that LPS induces an increase in AChE and a decrease in ChAT levels [58–60]. The present results may be attributed to the idea that the increase in AChE and the decrease in ChAT levels following LPS exposure result from the activation of inflammatory pathways, particularly via NF-κB signaling and inflammatory cytokines, which can hinder ACh synthesis [61, 62].

Additionally, the present data revealed that the LPS group showed a highly significant decrease in CAT and SOD levels and a highly significant increase in MDA levels in the brain hippocampal homogenate. These results are in consistent with different studies confirmed that the LPS administration increased the levels of MDA and disrupted the endogenous antioxidant levels, which was evident by decreased SOD and catalase [43, 63, 64].

A possible interpretation of these results aligns with the findings of Ayala et al. [65] and Essadek et al. [66], who demonstrated that LPS stimulates immune cells to produce ROS, leading to the release of inflammatory cytokines. This inflammatory response can result in increased lipid peroxidation and antioxidant depletion.

At histopathological level, hippocampal tissue of the LPS group showed severe histological changes in most areas of the CA1 region. These changes included significant vacuolization, reduction of the pyramidal cell layers to 2–3 layers of neurons with pyknotic nuclei, neuronal shrinkage, cellular atrophy, and the presence of some cells with irregularly shaped outlines. Additionally, there was clear gliosis in the SO and SR. These findings are in agreement with Bahaidrah et al. [67] and He et al. [68], who noticed that the histological effect of LPS on hippocampal tissue demonstrated the presence of neurodegeneration with darker neuronal cells than the control group. Also, there were cell shrinkage, darker and elongated-looking cells, and augmented apoptotic neurons. Our results could be attributed to the fact that LPS administration triggers an immune response that leads to several histological changes in the brain via microglial activation, which ends in the release of cytokines. These cytokines contribute to neuroinflammation and can lead to neuronal injury and death as well as synaptic disruption. This synaptic disruption is associated with neuronal cell damage as documented by He et al. [68]. Additional evidence has shown that LPS generates free radicals, leading to the peroxidative destruction of brain lipid membranes and subsequent severe brain damage, including necrosis [69]. Also, the present findings showed that LPS administration increased caspase-3 and IL-6 expression in the hippocampal tissues. Similarly, Beurel et al. [70] and Sangaran et al. [71] found that injecting LPS at specific concentrations increases the secretion of caspase-3, a critical executioner caspase implicated in apoptotic cell death, as well as an increase in IL-6 synthesis via neuroinflammatory conditions. The elevation in these values may be attributed to mechanisms involving TLR4 signaling, oxidative stress, and mitochondrial dysfunction. These pathways lead to apoptosis and inflammation resulting in significant histological and functional alterations in the brain tissue [14, 72].

As expected, the current findings for the LPS + CAP group revealed a significant increase in the discrimination ratio, ratio index, and % alternation. The present results are in agreement with the previously documented by Abrashei et al. [73] and Dong et al. [74], who suggested that the treatment with CAP, which acts as an ACE inhibitor, exerts improvement in behavioral tests. Additionally, Hou et al. [75] emphasized that CAP has a significant effect on reducing cognitive impairment and modulating behavior by reducing neuroinflammation, improving cognitive function, and mitigating the effects of neurodegenerative diseases, potentially offering therapeutic benefits in conditions such as epilepsy and AD.

Again, the present findings of the LPS + CAP revealed a significant reduction in the concentration of NF-κB, TNF-α, IL-1β, Aβ level, ACE and AChE activity, accompanied by a significant elevation of ChAT levels in the homogenate of the brain hippocampus. In line with the present study, Abareshi et al. [73] and Sahin et al. [76] indicated that the treatment with CAP decreased NF-κB concentration in the homogenate tissue. In addition, Asraf et al. [77] showed that RAS intervention by CAP ameliorated astrocytic and glial activation and reduced the production of TNF-α and IL-1β. Moreover, Bhat et al. [78] attributed the anti-inflammatory mechanism of CAP to the inhibition of pro-inflammatory cytokine production and NF-κB as a result of ACE’s reduction. Similarly, Mohapatra et al. [79] demonstrated that CAP can reduce Aβ-induced toxicity and improve cognitive functions through its anti-inflammatory effects. Furthermore, Tao et al. [80] revealed that CAP employs competitive inhibition to reduce ACE activity by attaching itself to the ACE’s active site, preventing angiotensin I from becoming angiotensin II. This reduction in angiotensin II levels leads to diminishing the inflammatory response mediated by angiotensin II in various tissues, including the brain. Also, the findings of the present study are in accordance with Arkhipov et al. [81] and Beheshti et al. [82], who revealed that CAP modulates the cholinergic system indirectly by reducing neuroinflammation as a result of decreasing angiotensin type II, which can impair acetylcholine synthesis and function. This creates a supportive environment for cholinergic neurons, enhancing their function, which plays a crucial role for learning, memory, and cognitive function.

Also, the LPS + CAP group revealed a significant increase in the CAT and SOD and a highly significant decrease in MDA in the homogenate of the brain hippocampus. These results are similar to those were previously explained by Abbassi et al. [83], Ali et al. [84], and Mohapatra et al. [79], who confirmed that CAP as a ACE inhibitor significantly improves cognitive function in LPS-induced dementia in rats by potentiating the antioxidant status of brain defense system by increasing the CAT and SOD content and decreased MDA production. Our observation could be attributed to the CAP ability to cross blood-brain barrier to decrease oxidative stress and increase antioxidant enzymes, modulation of inflammatory cytokines, and inhibition of the RAS as documented by Abareshi et al. [28], Abiodun et al. [85], and Messiha et al. [86].

The hippocampal tissues of the LPS + CAP group retained its normal appearance to some extent relative to the LPS group. The SP in the CA1 region appeared with 2–3 spaced layers, a decline in the pyknotic nuclei of the pyramidal cells, decreased vacuolization, and reduction of the proliferation of glial cells in both the SO and SR. These results are in good concern with several studies accomplished by Fazal et al. [87] and Mohapatra et al. [79], who revealed that neurodegeneration was fewer in CAP-treated rats, and it exhibited improved and organized neuronal glial cells in comparison to the LPS- administered group.

The present study could be attributes captopril’s neuroprotective effect to the opinion of Nestor et al. [88], Asraf et al. [77], and Abiodun et al. [85], who confirmed that CAP treatment reduced the number of damaged neurons in the hippocampus by modulation of the RAS and, as a result, ACE is reduced, which can influence cerebral blood flow and possibly affect brain tissue structure and decrease inflammatory responses in the brain, leading to improved histological changes.

Again, the current data demonstrated a moderate expression of caspase-3 and IL-6 in the LPS + CAP group compared to the LPS group. In the same line, Tzeng et al. [89] found that CAP controlled caspase-3 because it had neuroprotective effects via modifying apoptotic pathways, which are consistent with our findings. Our observation could be attributed to the explanation of Abareshi et al. [28], who confirmed that CAP has anti-inflammatory characteristics by reducing the release of inflammatory cytokines such as IL-6, hence alleviating neuroinflammation.

The current study focused on crocin, as a natural compound that alleviates LPS-induced behavioral, biochemical, histopathological, and immunohistochemical abnormalities associated with memory impairments. Behavioral tests are frequently engaged to estimate crocin’s effects on AD [90]. Our present data showed that the oral administration of CR in LPS + CR and LPS + CAP + CR groups showed a highly significant increase in behavioral tests. The present study is in consonance with the findings of Pitsikas and Tarantilis [91] and Radahmadi et al. [92], who postulated that CR mitigates the deleterious effect of LPS on memory impairment by elevating discrimination ratio, ratio index, and % alternation. In the same context, Singh et al. [93] and Mazumder et al. [24] clarified that the inhibitory effect of CR against learning and memory impairments in behavioral tests induced by LPS occurs through multiple mechanisms. The improvement in behavioral test performance following CR administration could be due to its neuroprotective and its anti-inflammatory properties leading to enhanced memory and learning abilities [94].

Interestingly, the present findings from the LPS + CR and LPS + CAP + CR groups revealed a highly significant reduction in the concentrations of NF-κB, TNF-α, IL-1β, Aβ, ACE, and AChE activity, along with a highly significant elevation of ChAT levels in the homogenate of the brain hippocampus. These observations are consistent with several studies demonstrated that CR modulates NF-κB levels in the brain by crossing the blood-brain barrier and reaching the central nervous system, thereby enhancing its therapeutic potential [94–96].

Additionally, studies by Sadoughi [97] and Haeri et al. [98] indicated that CR alleviates inflammation in AD by targeting multiple pathways, such as reducing pro-inflammatory cytokines like TNF-α and IL-1β. In line with these findings, the reduction in these parameters in our study can be interpreted according to the opinions of Hosseini et al. [99] and Samieri et al. [100], who reported that CR dampens the inflammatory response by several mechanisms, including blocking NF-κB translocation to the nucleus, regulating inflammatory mediators, and reducing microglial activation. Furthermore, crocin reduces the expression of pro-inflammatory markers such as TNF-α and IL-1β, which are elevated in LPS-treated models [27, 101].

Similarly, CR administration reduces Aβ accumulation due to its neuroprotective and antioxidant properties, as well as its positive effects on synaptic function [95, 102]. Additionally, CR attenuates Aβ accumulation at multiple levels, including regulating amyloid precursor protein (APP) cleavage to divert it from the amyloidogenic pathway, enhancing Aβ clearance through the stimulation of enzymes like neprilysin and autophagy pathways, and exerting anti-inflammatory effects [103].

In agreement with our findings, Ghasemnejad-Berenji [104] and Bastani et al. [105] documented that CR treatment reduces ACE levels and improves learning and memory. Similarly, Khalil et al. [106] demonstrated that CR alleviates ACE activity by blocking inflammatory pathways like NF-κB, which indirectly reduces ACE upregulation associated with inflammation. Crocin’s neuroprotective effects help restore balance in the renin-angiotensin system, thereby inhibiting ACE activity and reducing the production of angiotensin II.

Crocin’s ability to alter major cholinergic system components makes it a promising therapeutic treatment for illnesses associated with cholinergic dysfunction. Cr can reduce AChE and improve the level of ChAT [107], and it helps to maintain healthy ACh levels, which are essential for learning and memory [108]. The possible interpretation the present data proved that CR inhibits AChE activity and so increases synaptic availability. It can also influence cholinergic function by increasing ChAT expression or activity, which promotes the production of ACh. This helps to restore cholinergic neurotransmission and treats cognitive impairments [109, 110].

Again, our data showed that there were a highly significant increase in the CAT and SOD and a highly significant decrease in the MDA level in the LPS + CR and LPS + CAP + CR groups in the homogenate of the brain. Parallel with our results, several reports proved that CR increased CAT and SOD levels and also decreased the MDA level in the brain of treated groups [64, 108, 111]. The possible interpretation is provided by Farkhondeh et al. [111], who stated that crocin by scavenging the free radicals is capable of protecting the cells against oxidative stress damages. Also, more explanation is revealed by Salem et al. [112] who elucidated that CR enhances antioxidant enzyme levels, thereby decreasing oxidative stress markers like malondialdehyde. Also, CR acts as anti-inflammatory agent by inhibiting the expression of pro-inflammatory cytokines and enzymes, thereby reducing inflammation-induced oxidative stress. This reduction in inflammation contributes to lower MDA levels and increased antioxidant enzyme activities.

The histological examination of the hippocampal tissue in the LPS + CR group and the LPS + CAP + CR group exposed that the hippocampal construction reserved its normal appearance to a large extent compared with the LPS group hippocampus. The SP in the CA1 region displayed marked improvement, with neurons appearing with normal morphology. Furthermore, a normal distribution of glial cells and well-preserved blood capillaries were observed in the SO and SR, reflecting restored structural integrity. These results are in line with Salem et al. [112] and Hashemzaei et al. [113], who showed that after CR treatment, the hippocampal tissues appeared without any neuronal loss, with good glial cells distribution and a healthy structure to blood capillaries.

As well as, in the current study, caspase-3 and IL-6 in the CR groups (LPS + CR and LPS + CAP + CR) showed weak expression in hippocampal tissues compared with the LPS group. Similar observations were made by D’amelio et al. [114] and Dastan et al. [115], who observed that CR can decrease caspase-3 expression and reduce levels of IL-6, which is convoluted in neuroinflammation. We attribute the reduction of caspase-3 and IL-6 according to the opinion of Shahbaz et al. [116] and Yang et al. [117], who emphasized that crocin, has anti-apoptotic and anti-inflammatory properties.

The possible elucidation of the neuroprotective effects of CR that are displayed in the present study is consistant with Dastan et al. [115], who demonstrated that the protective effects of CR against LPS-induced histological changes in the brain occur through several mechanisms, such as anti-inflammatory effects, antioxidant properties, reduction of oxidative stress, and anti-apoptotic activity, all of which help to restore the normal appearance of the hippocampus. Also, this action of CR was explained by Shahidani et al. [118], who stated that crocin can stimulate biological activity that may enhance developmental and hippocampal neurogenesis and neuronal repair.

## Conclusion

The present study demonstrated that LPS exposure activates NF-κB signaling and increases pro-inflammatory cytokines, thereby influencing Aβ production through NF-κB and ACE activity. LPS also impairs cholinergic neurons and promotes oxidative stress, accelerating memory deficits, while histological and immunohistochemical analyses confirmed hippocampal damage and increased immunostaining. In contrast, CR exhibited multi-target ameliorative mechanisms by attenuating NF-κB activation, reducing inflammatory cytokines, decreasing ACE activity, and enhancing both Aβ clearance and cholinergic function. Furthermore, CR improved the antioxidant defense system by modulating SOD and CAT activity, restoring the balance between oxidative stress and antioxidant capacity. Importantly, this study highlights crocin’s multifaceted role in improving behavioral outcomes and mitigating neuroinflammation, oxidative stress, and histological alterations, all of which are critical in neurodegenerative conditions. These findings not only expand the current understanding of crocin’s mechanisms but also suggest its possible translational value as a natural therapeutic candidate for oxidative stress–related neurodegenerative disorders. While the study presents compelling evidence for crocin’s protective effects, it is essential to consider that the complexity of neurodegenerative diseases may require multifactorial approaches beyond single compounds like crocin for effective treatment strategies.

## Data Availability

This article contains all the data that was created or evaluated during the research.

## Abbreviations

LPS: lipopolysaccharide
CAP: captopril
CR: crocin
NF-κB: nuclear factor kappa B
TNF-α: tumor necrosis factor-alpha
IL-1β: interleukin-1Beta
Aβ: amyloid beta
ACE: angiotensin converting enzyme
AChE: acetyle cholinesterase
ChAT: choline acetyle transferase
CAT: Catalase
SOD: superoxide dismutase
MDA: malondialdehyde
